# A55 YOU ARE WHAT YOUR GREAT, GREAT GRANDPARENTS PARENTS ATE: PRE-NATAL GLYPHOSATE EXPOSURE INDUCES DYSBIOSIS, METABOLIC DYSFUNCTION AND BEHAVIOURAL ABNORMALITIES THREE GENERATIONS AFTER EXPOSURE

**DOI:** 10.1093/jcag/gwac036.055

**Published:** 2023-03-07

**Authors:** J A Barnett, J K Josephson, M L Bandy, N Haskey, J Gibon, H Chiang, D L Gibson

**Affiliations:** 1 Biology; 2 Medicine , University of British Columbia, Kelowna, Canada

## Abstract

**Background:**

Canada is one of the most prolific users of the herbicide glyphosate (tradename Roundup®), with over 25 million kilograms purchased annually. Glyphosate is commonly applied pre-harvest as a desiccant, leading to higher residues in many foods consumed within Canada, including wheat, cereals, legumes, and soy products. Glyphosate prevents the synthesis of aromatic amino acids by inhibiting the Shikimate pathway, which, besides killing weeds, can also inhibit bacterial growth and promote bacterial dysbiosis. Dysbiosis has been associated with multiple disease states, including intestinal inflammation and metabolic dysfunction, which both include anxiety, depression, and cognitive dysfunction as co-morbidities.

**Purpose:**

My goal is to clarify if there are harmful glyphosate effects on the gut bacteriome, resulting in altered metabolic health, damaging intestinal inflammation, and behaviour changes through the gut-brain axis. We explored two environmentally relevant doses throughout our experiments: (1) the acceptable daily intake currently set by the Environmental Protection Agency (EPA) (1.75mg/kg body weight/day); (2) the North American dose calculated with a registered dietician based on literature values of glyphosate within items that make up a typical Canadian diet (0.01mg/kg body weight/day.)

**Method:**

Breeding pairs consisting of Muc2^-/-^ (colitis susceptible) and Muc2^+/-^ (littermate control) mice were exposed to glyphosate during pregnancy. A subset of each generation underwent behavioural testing to characterize anxiety, memory, and locomotor activity. Animals also underwent metabolic testing, including oral glucose and insulin tolerance tests. Only the parental generation of animals received glyphosate. In total, three generations of animals were raised.

**Result(s):**

Our results show that healthy mice whose great-grandparents were exposed to glyphosate at levels currently deemed safe by the EPA exhibited decreased locomotor activity. Furthermore, mice whose parents were exposed to either North American or EPA glyphosate levels exhibit symptoms of metabolic dysfunction in healthy and colitis-susceptible mice, including higher fasting blood glucose, an inability to efficiently clear glucose and impaired insulin response. Additionally, Muc2^-/-^ mice whose parents were exposed to levels of glyphosate found within the North American diet during pregnancy showed significant impairments in working memory.

**Conclusion(s):**

This study is the first to highlight the transgenerational effects of glyphosate at levels previously deemed safe for human exposure on locomotor activity, working memory and metabolism. Additionally, this study highlights how environmental toxins within our food system, such as glyphosate, may play a vital role in the etiology of many diseases in healthy and colitis-susceptible populations by promoting a sedentary lifestyle, metabolic dysfunction, and behavioural deficits.

**Please acknowledge all funding agencies by checking the applicable boxes below:**

CAG, CCC, Other

**Please indicate your source of funding;:**

NSERC

**Disclosure of Interest:**

None Declared

